# Membrane-Focused Strategies Against *Acinetobacter baumannii*: The Therapeutic Potential of Functional Copolymers

**DOI:** 10.3390/antibiotics15040408

**Published:** 2026-04-17

**Authors:** Barbara Cardoso Domingues, Marc Maresca, Jean-Michel Bolla, Véronique Sinou

**Affiliations:** 1 Aix Marseille Université, INSERM, SSA, MCT, 13385 Marseille, France; barbara.cardoso-domingues@etu.univ-amu.fr; 2 Aix Marseille Univ, CNRS, Centrale Marseille, iSm2, 13013 Marseille, France; m.maresca@univ-amu.fr

**Keywords:** antimicrobial resistance, *Acinetobacter baumannii*, membrane-targeting copolymers, multidrug-resistant (MDR) pathogens

## Abstract

Antimicrobial resistance is a serious global public health concern, with *Acinetobacter baumannii* recognized as one of the most problematic multidrug-resistant (MDR) pathogens. This Gram-negative bacterium is highly persistent in the environment, possesses a remarkably adaptable cell envelope, and forms biofilms. As the effectiveness of conventional antibiotics declines, alternative strategies are being actively explored, particularly membrane-targeting approaches based on synthetic copolymers. These compounds mimic antimicrobial peptides, offer enhanced stability and structural tunability, and have a lower propensity to develop resistance. Recent advances in polymer chemistry have led to the design of antibacterial polymers with activity against MDR *A. baumannii*. Some of these act synergistically with existing antibiotics, restoring bacterial susceptibility or disrupting biofilms. However, their non-degradability remains a concern due to its potential implications for body/environment accumulation and related toxicity and/or selection of resistant strains. This review examines the biology of the *A. baumannii* cell envelope, its resistance mechanisms, and treatment limitations, while emphasizing the promise of membrane-active copolymers. By bridging materials science and microbiology, these approaches offer promising strategies for combating World Health Organization priority pathogens.

## 1. Introduction

Over the past 50 years, the life expectancy of the world’s population has increased substantially, largely due to the introduction of antibiotics, which have significantly reduced mortality from infectious diseases. However, their widespread use in community healthcare, hospital settings, and livestock farming has exerted substantial selective pressure on bacterial populations [1]. This pressure has led to the increasingly rapid emergence of antibiotic-resistant strains, which in some cases have appeared within the first year of a new antibiotic’s introduction [2]. As a result, bacterial infections have become the second leading cause of death worldwide.

In 2021, 4.7 million deaths were associated with antimicrobial resistance (AMR), with 1.4 million deaths attributable to bacterial AMR. Based on the latest forecasts, AMR could be linked to 8.22 million global deaths per year, of which 1.91 million would be directly associated with AMR infections by 2050 [3]. The situation is of particular concern for seven bacterial species—*Enterococcus*, *Staphylococcus*, *Klebsiella*, *Acinetobacter*, *Pseudomonas*, *Enterobacter* sp., and *Escherichia coli*—grouped under the acronym ESKAPEE [4].

**Figure 1:**
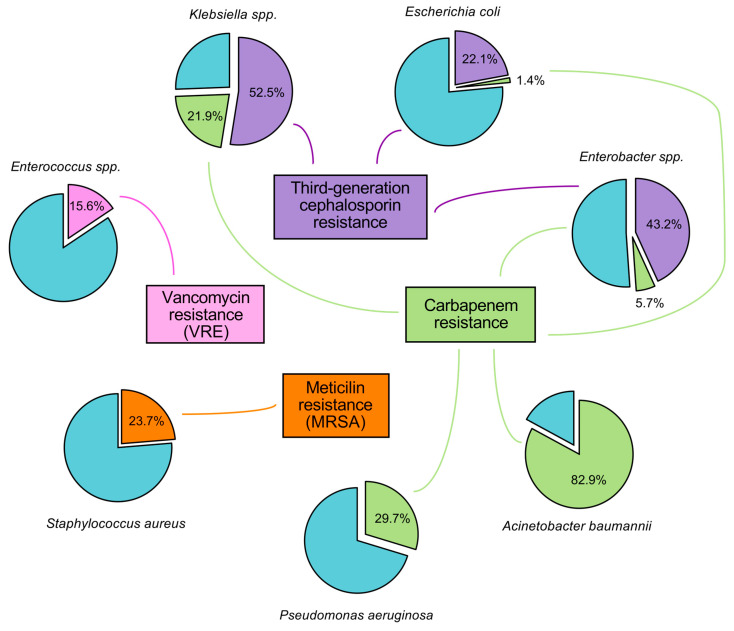
Prevalence of critical AMR phenotypes across key bacterial pathogens.

*A. baumannii* with *S. aureus*, *E. coli*, *K. pneumoniae*, and *P. aeruginosa* is among the bacterial pathogens with the highest AMR-attributable mortality, each accounting for at least 100,000 deaths in 2021 [3]. Furthermore, methicillin-resistant *S. aureus* (MRSA), carbapenem-resistant *K. pneumoniae,* and carbapenem-resistant *A. baumannii* (CRAB) are identified among the four pathogen–drug combinations that saw an increase of more than 25,000 attributable deaths per year between 1990 and 2021 (Figure 1). Among them, *A. baumannii* illustrates particularly well this alarming trend. In just four decades, it has shifted from a low-priority pathogen—characterized by limited virulence and susceptibility to most antibiotics available at the time—to a major MDR threat, often pioneering novel resistance mechanisms [5]. Colistin and tigecycline remain the only antibiotics active against MDR *A. baumannii* and are considered treatments of last resort. However, colistin-resistant strains have been increasingly reported worldwide [6].

**Figure 2:**
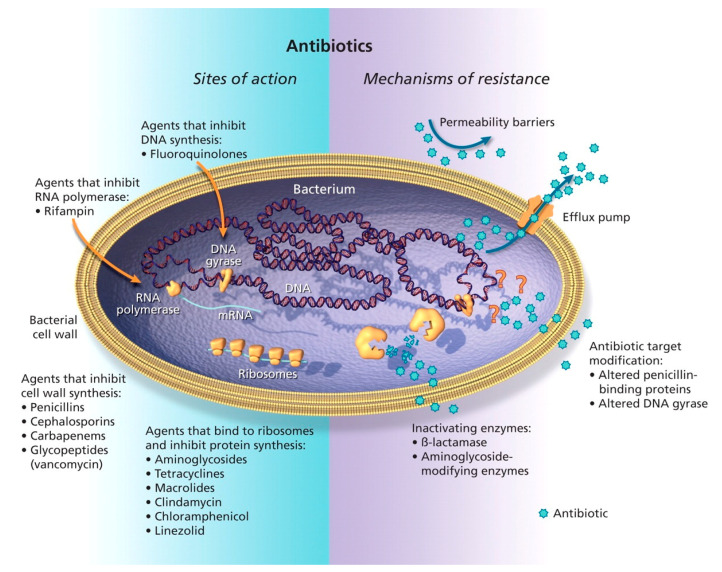
Overview of primary antibiotic targets and associated bacterial resistance mechanisms, figure from [8]. The diagram illustrates the sites of action for major antibiotic classes (left panel). Key targets include, among others, the inhibition of cell wall synthesis (e.g., β-lactams), protein synthesis (e.g., aminoglycosides, tetracyclines), and nucleic acid synthesis (e.g., fluoroquinolones). The right panel depicts prominent resistance mechanisms; among these are (1) restricted permeability of the outer membrane (OM) barrier, (2) active expulsion by efflux pumps, (3) enzymatic inactivation of the antibiotic, and (4) target site modification or protection.

There are numerous mechanisms through which antibiotic resistance develops. The most documented include alterations at target sites, inactivation by specific enzymes, changes in membrane permeability, and expression of efflux pumps, among others, as represented in Figure 2 [7].

However, bridging the gap between our understanding of resistance mechanisms and the availability of effective treatments remains a major hurdle, as reflected in the current state of antibiotic development [9]. The global clinical pipeline for antibacterial agents includes 90 therapeutic entities or combinations targeting World Health Organization (WHO) Bacterial Priority Pathogens, or *Helicobacter pylori* [4,10]. This count is divided into 50 traditional and 40 non-traditional agents. Of the 50 traditional antibacterial agents currently in clinical development, 25 (50%) are considered potentially innovative based on the WHO criterion of showing no known cross-resistance; among these, 14 demonstrate activity against WHO Critical Priority Pathogens [11]. To address these priority pathogens, the current development pipeline includes several candidates with innovative features that extend beyond mere structural modifications of existing antibiotics and are effective against the difficult-to-treat CRAB (Table 1).

Among these promising agents, several stand out for their novel mechanisms and potential to overcome resistance (Table 1). For example, BWC0977 is a Novel Bacterial Topoisomerase Inhibitor (NBTI) that shows no cross-resistance with current antibiotics and works by inhibiting DNA gyrase and topoisomerase IV [23]. Similarly, RECCE 327 (R327) is a fully synthetic polymer with a unique mechanism of action that disrupts bacterial energy production [51]. Xeruborbactam is a newly developed bicyclic boronate BLI β-lactamase inhibitor, representing a new chemical class, designed to inhibit a broad spectrum of β-lactamases, including the challenging Class B metallo-β-lactamases (MBLs) and Class D carbapenemases (OXA-23 in *A. baumannii*). This innovation aims to restore the effectiveness of partnered β-lactam antibiotics [75].

Other innovative approaches include Zosurabalpin, a macrocyclic peptide that targets a unique pathway by inhibiting lipopolysaccharide (LPS) transport—an essential process for Gram-negative cell membrane formation [72]—and OMN6, a synthetic cyclic peptide engineered to disrupt bacterial membranes directly [76].

As illustrated in Table 1, membrane-targeting compounds are at the forefront of antibacterial drug discovery. By focusing on the bacterial membrane, these agents bypass the need to cross the plasma membrane, making them less vulnerable to resistance mechanisms related to membrane permeability and efflux. In addition, their action on the membrane allows them to (i) rapidly kill bacteria (within seconds/minutes, in contrast to other bactericidal antibiotics acting in hours), preventing bacterial division and thus limiting the risk of resistance development, and (ii) be active against bacteria already resistant to other types of antibiotics targeting intracellular enzymes/processes. This approach is particularly relevant given the growing scientific attention to *A. baumannii*, as bibliometric trends reveal a sustained increase in publications [77,78].

**Figure 3:**
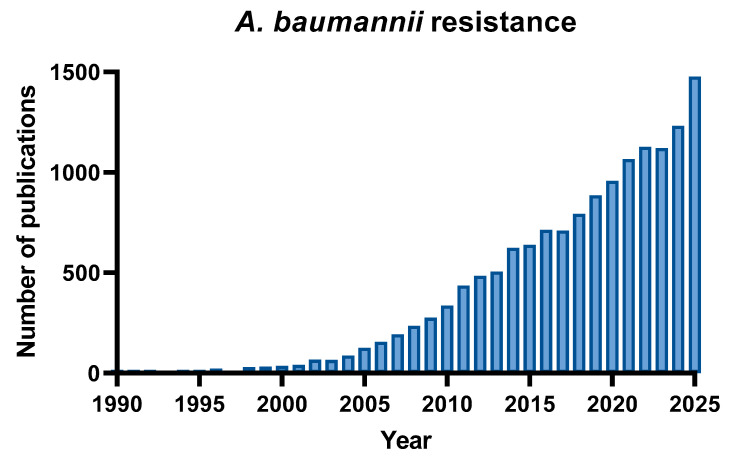
Bibliometric evolution of research on *A. baumannii* resistance (1990–2025). The bar chart illustrates the annual number of publications indexed in PubMed using the search query “*A. baumannii* resistance”. The exponential growth, particularly the surge in publications exceeding 1000 per year post-2021, underscores the increasing global health priority of this pathogen.

By searching “*A. baumannii* resistance” in PubMed, the number of publications increased from less than 50 articles between 1990 and 2001 to over 500 per year after 2013, and over 1000 since 2021, reflecting the global spread of carbapenem- and pan-drug-resistant isolates (Figure 3).

Accordingly, this review provides an in-depth overview of the unique architecture of the *A. baumannii* cell envelope, which acts as the primary line of defense against conventional antibiotics, and highlights the potential of membrane-active copolymers as a promising therapeutic strategy.

## 2. The Pathogen: *A. baumannii*’s Arsenal—Membrane and Beyond

### 2.1. Determinants of Colonization and Pathogenicity

*A. baumannii*, a Gram-negative pathogen, uses a complex set of virulence factors that support its remarkable ability to survive in hostile environments and resist antimicrobial therapy [79]. These features work together to enhance its pathogenicity and persistence in clinical settings, and include outer membrane (OM) porins, the biofilm-associated protein, phospholipases, LPS/LOS (lipopolysaccharide/lipooligosaccharide), proteases, advanced iron acquisition systems, penicillin-binding proteins (PBPs), and various protein secretion systems.

**Figure 4:**
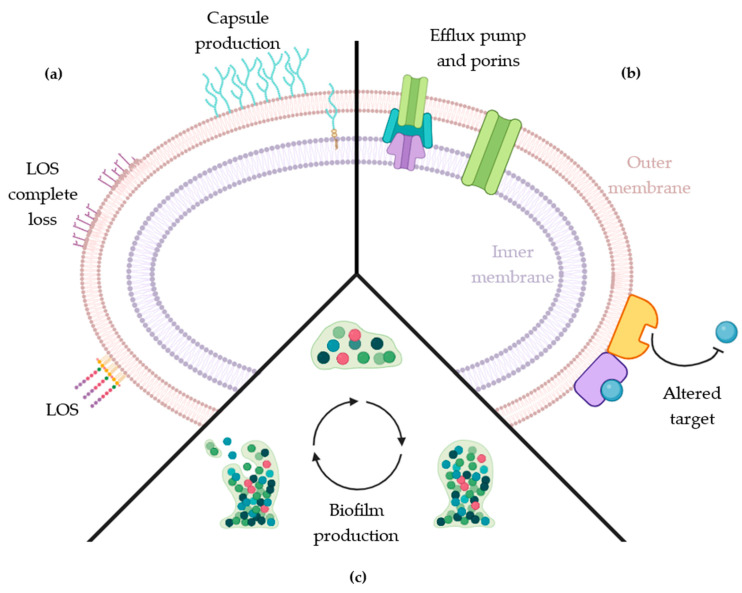
Mechanisms of AMR in *A. baumannii*. (**a**) Outer membrane modifications: Structural changes that physically block drug access, including the complete loss of LOS, alterations to its structure (LOS), and the formation of a protective capsule. (**b**) Target/Efflux mechanisms: decreased intracellular drug concentration through efflux pumps (actively removing drugs) and changes in outer membrane porins (reducing influx). Resistance can also occur through altered drug targets (such as modified binding sites) within the cell. (**c**) Community lifestyle: the formation of biofilms, a community enclosed in a matrix, which creates physical and physiological barriers, leading to reduced drug penetration and a state of metabolic tolerance that protects embedded bacteria from antibiotics.

Among these virulence factors, the cell envelope is the most critical defensive structure serving both as a physical barrier and as a platform for resistance mechanisms (Figure 4). Beyond the basic cell wall, *A. baumannii* employs additional protective layers, including a polysaccharide capsule, that functions as an outer shield against environmental stresses and host defenses. Its defensive capacity is further enhanced when the bacteria adopt a community lifestyle, forming highly resistant biofilms that confer additional protection.

Beyond physical persistence, *A. baumannii* utilizes sophisticated mechanisms to scavenge iron from host cells, which is essential for its survival and virulence. The bacterium is reported to produce three important siderophores: acinetobactin, baumannoferrin, and fimsbactin. Among these, acinetobactin exerts a major influence on pathogenesis, as it possesses a high-affinity catechol hydroxamate structure that effectively competes with host proteins for iron. This iron-acquisition capability is considered a critical virulence factor, enabling the pathogen to thrive in the nutrient-restricted environment of the host [80].

### 2.2. A. baumannii Cell Wall

The multilayered cell envelope of *A. baumannii* is key to its resilience, virulence, and inherent resistance mechanisms. As depicted in recent structural overviews, this envelope serves as a scaffold for a diverse arsenal of virulence factors, including the polysaccharide capsule, outer membrane proteins (OMPs), and secretion systems, which collectively orchestrate the pathogen’s defense and interaction with the host [81].

Among these components, OMPs constitute a critical functional interface between the bacterium and its environment, playing essential roles in nutrient uptake, membrane stability, and virulence [82]. Key OMPs identified in this pathogen include BamA, LptD, Omp33–36, and OmpW.

BamA is indispensable for OM biogenesis, mediating the assembly and insertion of other OMPs. LptD is required for the transport of LPS to the outer leaflet of the OM. Omp33–36 functions as a porin that facilitates the diffusion of small hydrophilic molecules, whereas OmpW forms a hydrophobic channel that is involved in iron homeostasis.

Among these proteins, OmpA (Outer membrane protein A) stands out as the most abundant and multifaceted surface protein, conferring a “two-pronged defense” essential for the pathogen’s success: it maintains the structural integrity of the cell envelope while simultaneously driving pathogenesis [83]. Structurally, OmpA acts as a slow, specific porin that spans the OM and plays a vital role in regulating permeability and stability under varying environmental conditions [84]. Regarding pathogenicity, OmpA is implicated in several key mechanisms. First, it is essential for bacterial adherence to host epithelial cells and promotes robust biofilm formation on abiotic surfaces, a critical factor for persistence in hospital environments [85]. Second, it actively facilitates the invasion of epithelial cells, functioning as a key invasin [86]. Finally, OmpA contributes to immune evasion by inducing host–cell apoptosis via mitochondrial targeting, thereby compromising the epithelial barrier [87]. A crucial factor in the intrinsic resistance of *A. baumannii* is the extremely low permeability of the OM, which is structurally up to 100 times less permeable than that of *E. coli* [88]. This low permeability greatly limits antibiotic entry, strengthening the effectiveness of its resistance mechanisms, and is not merely passive but results from a specific evolutionary adaptation: the paucity of large, non-specific porins. In typical *Enterobacter* sp., the influx of antibiotics is facilitated by abundant, large-channel non-specific porins such as OmpF and OmpC [89]. In contrast, *A. baumannii* lacks homologs of these wide channel porins. Instead, its OM is populated by “slow” porins, predominantly OmpA, and a diverse array of specific, substrate-gated channels. Among these, CarO (Carbapenem-associated outer membrane protein) and OprD play pivotal roles. CarO, for instance, is not a continuously open pore; it functions as a specific channel for the uptake of basic amino acids and possesses a gated loop region that restricts the passive diffusion of non-cognate molecules [88]. Consequently, the entry of small hydrophilic antibiotics, including β-lactams and saccharides, is severely restricted. This low permeability creates a synergistic effect with intracellular resistance mechanisms. By limiting the rate of antibiotic influx, the bacterium allows even low-level expression of efflux pumps (such as the AdeABC RND system [90] or periplasmic β-lactamases to effectively clear the drug before it reaches its lethal target concentration. Thus, the structural “tightness” of the OM acts as a force multiplier for all other resistance mechanisms [5].

**Figure 5:**
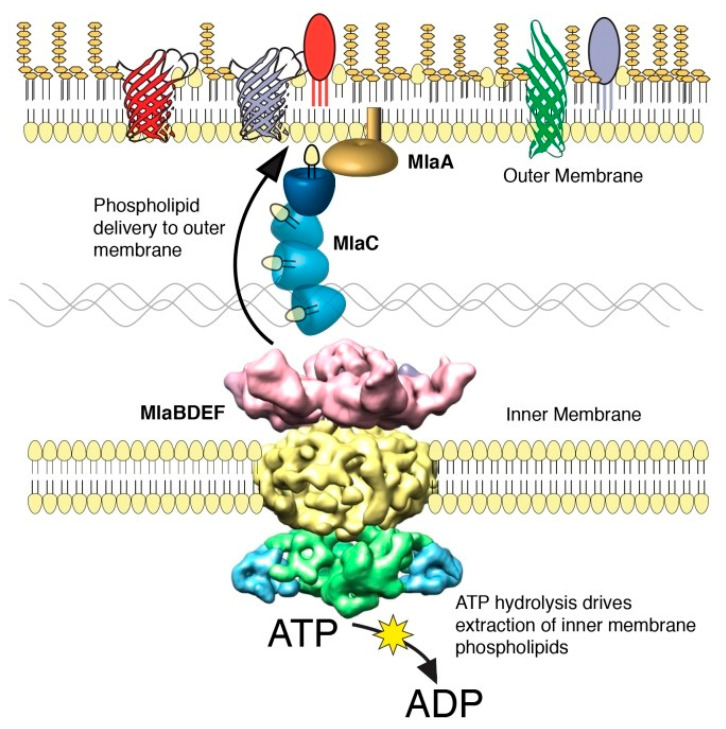
Model of phospholipid transport by the Maintenance of lipid Asymmetry (Mla) system in *A. baumannii*. The Mla system maintains outer membrane stability via an anterograde phospholipid transport pathway. The inner membrane ABC transporter MlaFEDB extracts newly synthesized phospholipids using energy from ATP hydrolysis and transfers them to the periplasmic chaperone MlaC. MlaC shields the hydrophobic lipid tails and ferries the cargo across the periplasm to the MlaA complex in the outer membrane, which facilitates their insertion into the membrane leaflets, thereby ensuring barrier integrity. Adapted from Kamischke et al., 2019 [93].

The barrier function of the OM is strictly dependent on its unique asymmetric architecture. In a healthy Gram-negative bacterium, the inner leaflet of the OM is composed of phospholipids (PLs), while the outer leaflet is exclusively populated by glycolipids (LPS or LOS). This trans-bilayer lipid asymmetry is crucial [91]. To maintain the barrier integrity, *A. baumannii* relies on the Mla (Maintenance of lipid Asymmetry) system (MlaFEDB-C-A), a highly conserved but non-canonical ATP-binding cassette (ABC) transporter complex that spans the entire cell envelope (Figure 5) [92,93].

The mechanism of the Mla system in *A. baumannii* has been characterized as an anterograde phospholipid transport pathway, essential for OM biogenesis and stability. In this model, the process begins at the inner membrane, where the ATP-binding cassette transporter MlaFEDB extracts newly synthesized phospholipids and transfers them to the periplasmic chaperone MlaC. MlaC then shields the hydrophobic lipid tails from the aqueous periplasmic environment and ferries the cargo across the periplasm to the OM complex. Finally, the MlaA lipoprotein, anchored in the OM, receives the phospholipids from MlaC and facilitates their insertion into the membrane leaflets. Disruption of this transport system compromises the OM barrier by preventing the proper delivery of bulk phospholipids required to maintain membrane density, leading to increased susceptibility to antibiotics and attenuated virulence [93].

The stability of the envelope is further reinforced by the tightly coordinated biogenesis of its various layers. A critical node in this network is the undecaprenyl pyrophosphate (Und-PP) synthase. Und-PP serves as the essential lipid carrier (a “shuttle”) for the soluble precursors of peptidoglycan, wall teichoic acids (WTA), and capsular polysaccharides. Without sufficient Und-PP, the bacterium cannot synthesize its cell wall or capsule. Recent genomic and functional studies have revealed a genetic and functional synergy between Und-PP biosynthesis and the Mla system [92]. It is hypothesized that defects in lipid asymmetry (Mla mutants) induce envelope stress that requires compensatory strengthening of the cell wall. The Und-PP synthase works in concert with the Mla system to ensure that the peptidoglycan mesh is robust enough to withstand turgor pressure even when the OM is stressed. This coordination is vital for *A. baumannii* to survive the “membrane stress” induced by host immune factors or antibiotics [94].

**Figure 6:**
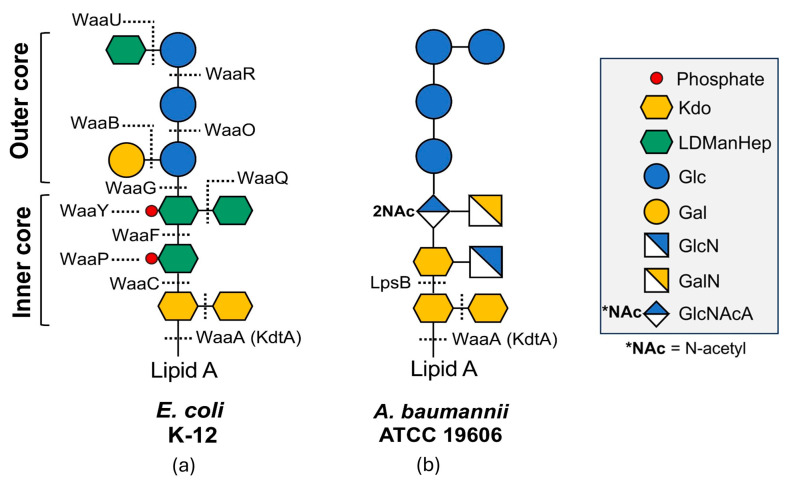
Comparative structural organization of surface glycolipids in *E. coli* versus *A. baumannii*, adapted from [98]. (**a**) Structure of the lipopolysaccharide (LPS) core from *E. coli* K-12. The schematic highlights the extensive waa-encoded enzymatic pathway (e.g., WaaA, WaaC, WaaQ) responsible for assembling the inner and outer core. Note the presence of phosphate groups (red dots) on the inner core heptoses, which contribute to membrane charge. (**b**) Structure of the LOS from *A. baumannii* ATCC 19606. This structure is synthesized by a distinct set of enzymes, including LpsB, and differs by the absence of core lateral phosphorylation and a specific sugar composition (Galactose, Glucose, GlcNAc). Although *A. baumannii* LOS lacks the long O-antigen chains found in smooth *Enterobacter* sp., the core oligosaccharide itself forms a dense, cross-linked barrier stabilized by divalent cations interacting with Kdo and lipid A residues.

While the OM of *Enterobacter* sp., such as *E. coli*, is typically characterized by LPS featuring a conserved core and variable O-antigen extensions, *A. baumannii* (strain ATCC 19606) presents a distinct surface topology dominated by LOS. As illustrated in the comparative structural analysis (Figure 6), distinct biosynthetic pathways govern the assembly of these glycolipids. In the *E. coli* K-12 model (Panel A), core assembly involves a complex cascade of glycosyltransferases encoded by the *waa* gene cluster (WaaA, WaaC, WaaF), which incorporates phosphate residues at specific sites (indicated by red dots). In contrast, the *A. baumannii* ATCC 19606 structure (Panel B) reveals a distinct composition. While it shares the WaaA (KdtA) enzyme for attaching Kdo residues to lipid A, the subsequent extension relies on specific transferases such as LpsB. Structurally, this LOS core lacks the specific heptose-phosphate modifications seen in *E. coli*. Instead, the membrane stability relies heavily on the electrostatic interactions between divalent cations (Mg^2+^, Ca^2+^) and the negatively charged carboxyl groups of the Kdo residues and the lipid A phosphates.

This compact structure is inherently resistant to detergents and hydrophobic antibiotics but renders the bacterium susceptible to chelating agents, which strip the stabilizing ions and destabilize the membrane [95,96,97].

The anchor of the LOS molecule, lipid A, forms the bioactive core of the OM. In *A. baumannii*, lipid A exists primarily in two major forms: a hepta-acylated variant (AB-A) and a hexa-acylated variant (AB-B). Both forms share a conserved backbone consisting of a β-(1→6)—linked D-glucosamine (GlcN) disaccharide that is phosphorylated at the 1- and 4′-positions.

**Figure 7:**
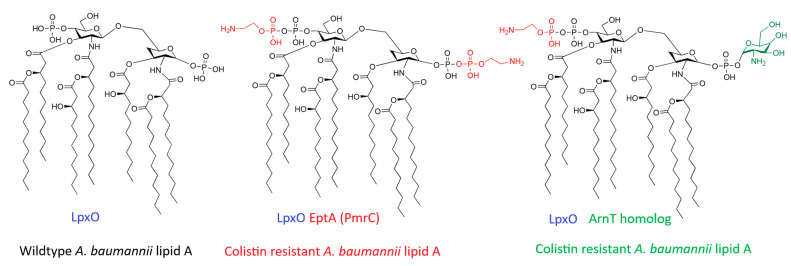
Molecular mechanisms of colistin resistance mediated by lipid A modifications in *A. baumannii*, adapted from [103]. (**Left**): Structure of wild-type lipid A (typically hepta-acylated), which is phosphorylated and negatively charged. (**Center**): Modified lipid A structures expressed by resistant strains. Activation of the PmrCAB two-component system induces the addition of phosphoethanolamine (pEtN) (shown in red) to the phosphate groups, catalyzed by the phosphoethanolamine transferase EptA (PmrC). (**Right**): Alternatively, glycosylation with galactosamine (shown in green) can occur via an ArnT homolog. These additions of positively charged groups neutralize the net negative charge of the membrane, electrostatically repelling cationic polymyxins.

The hepta-acylated lipid A is particularly distinct compared to the canonical hexa-acylated lipid A of *E. coli* [99]. This degree of acylation contributes to a more hydrophobic and stable membrane bilayer, influencing both immunogenicity and interactions with cationic antimicrobial peptides, including the last-resort antibiotics: polymyxins (colistin and polymyxin B) [99,100]. Polymyxins are cationic peptides that kill bacteria by binding to the negatively charged phosphate groups (at the 1- and 4′-positions) of lipid A [100]. They displace the stabilizing divalent cations and insert their fatty acid tail into the membrane, causing disruption and lysis. Resistance to colistin in *A. baumannii* is rarely plasmid-mediated but rather arises from chromosomal mutations in the PmrCAB two-component regulatory system [101]. Under conditions of stress (low Mg^2+^ or exposure to cationic peptides), the sensor kinase PmrB phosphorylates the regulator PmrA [101]. Phosphorylated PmrA upregulates the expression of the *pmrC* gene (also known as *eptA*) and the *naxD* gene [101,102]. PmrC is a phosphoethanolamine transferase that catalyzes the addition of phosphoethanolamine (pEtN) residues to the 1′ or 4′ phosphate groups of lipid A (Figure 7). Additionally, NaxD is a deacetylase involved in the synthesis of galactosamine (GalN), which can also modify lipid A phosphate groups [102].

While lipid A modification is the predominant mode of resistance, *A. baumannii* possesses a nearly unique capability among Gram-negative pathogens: it can develop high-level colistin resistance through the complete loss of LOS production [104]. This radical mechanism arises from spontaneous mutations in the genes encoding the first three enzymes of the lipid A biosynthetic pathway (*lpxA*, *lpxC*, or *lpxD*). The inactivation of one of these genes results in the total absence of lipid A, thereby completely removing the binding target for polymyxins. Although this confers resistance, the loss of LOS incurs a significant biological fitness cost, including reduced growth rate, attenuated virulence, and increased susceptibility to other antibiotics and host innate immune factors, which explains why this phenotype is less frequently observed in clinical isolates compared to PmrCAB-mediated modifications [104].

### 2.3. Capsule Formation and Its Protective Role

Like some other pathogens, *A. baumannii* produces a capsular polysaccharide (CPS) that surrounds its OM [105]. This structure primarily composed of repeating oligosaccharide units, plays a critical role in several biological processes and is a major determinant of virulence [106]. The structural diversity of CPS is genetically encoded by the highly variable K locus (KL), which contributes to extensive antigenic heterogeneity among clinical isolates. At the molecular level, CPS biosynthesis and assembly predominantly occur via the Wzy-dependent pathway, which is tightly regulated by the *wza-wzb-wzc* operon. This system controls both polysaccharide polymerization and export. For example, in the strain AB307-0294, K1 capsule polymerization and assembly re-quire the presence of two genes that are functional homologs of key components of the *wza–wzb–wzc* system: *ptk*, encoding a putative protein tyrosine kinase, that regulates capsule chain length and thickness, and *epsA*, encoding a putative OM polysaccharide export protein that forms a multimeric channel for polymer translocation [107]. These genes are conserved across various *A. baumannii* strains, highlighting their fundamental role in capsule assembly and export. Capsules fundamentally act as protective barriers that confer resistance to complement- and phagocyte-mediated killing, thereby promoting evasion of the innate immune response. In parallel, it also protects a wide range of external stresses, including desiccation, disinfectants, and certain antimicrobial agents [79].

This protective effect is largely attributable to the biophysical properties of the capsule. While it promotes survival in human serum by reducing complement deposition, the dense and highly hydrated polyanionic matrix forms a steric and electrostatic barrier that acts as a molecular “cloak” that masks and conceals pathogen-associated molecular patterns, such as the LOS and OMPs, from host recognition systems. As a result, activation of the alternative complement pathway is impaired, notably through the inhibition of C3b deposition on the bacterial surface, which is essential for opsonization and downstream phagocytosis [107,108].

Beyond this passive shielding role, the capsule is increasingly recognized as a dynamic and tightly regulated determinant of pathogenicity. Variations in capsular thickness directly influence bacterial fitness during infection. Hypercapsulated phe-notypes, frequently observed in CRAB strains with a hypermucoid appearance, are associated with increased virulence and enhanced tolerance to antibiotics [108]. Mechanistically, a thicker capsule not only restricts the diffusion of large or hydrophobic antimicrobial molecules but also reflects an active stress adaptation response. Indeed, exposure to antibiotics can activate the BfmRS two-component regulatory system, a global signaling pathway that controls envelope homeostasis. Activation of BfmRS induces a strong transcriptional upregulation of genes within the K locus, resulting in stress-induced hypercapsulation that reinforces the physical barrier of the cell envelope and limits further drug influx [109].

However, this protective strategy involves important trade-offs. While increased capsule production enhances immune evasion and environmental resilience, it can negatively impact bacterial interactions with surfaces. The bulky capsular layer masks key surface structures, including adhesins and the Type IV pilus machinery, which are essential for attachment and horizontal gene transfer. Consequently, reduced capsulation can favor surface adhesion, biofilm initiation, and natural transformation efficiency, highlighting a finely tuned balance between virulence, persistence, and adaptability in *A. baumannii* [110].

While this dense barrier enhances survival in human serum by preventing complement deposition, its immune-evasion capability is largely attributable to biophysical shielding.

The dense, polyamionic capsular layer acts as a molecular cloak that physically masks pathogen-associated molecular patterns, such as the LOS and the OM proteins. This prevents recognition by the host and severely impairs the alternative complement pathway by specifically blocking the deposition of C3b on the bacterial surface.

### 2.4. Biofilm Formation and Resistance

Several studies on infectious diseases have demonstrated a strong association between biofilm formation and the pathogenesis of *A. baumannii*. Approximately 70% of bacterial infections are biofilm-mediated and primarily associated with medical devices such as ventilators and catheters [111]. This sessile lifestyle confers a state of “recalcitrance” enabling bacteria to withstand antibiotic concentrations 10 to 1000 times higher than those effective against planktonic cells [112]. This heightened tolerance is largely attributed to the extracellular polymeric matrix that characterizes biofilms. Biofilms are structured microbial communities embedded within a self-produced matrix primarily composed of exopolysaccharides, proteins, and extracellular DNA (eDNA), which together provide physical protection against antimicrobial agents. In *A. baumannii*, the biofilm matrix is predominantly composed of Poly-β-(1,6)-N-acetylglucosamine (PNAG), synthesized by the PgaABCD machinery, which is essential for bacterial aggregation and adhesion to abiotic surfaces [113]. In addition, eDNA acts synergistically with PNAG as a structural scaffold, further reinforcing the biofilm architecture and limiting antibiotic penetration, particularly for positively charged aminoglycosides such as gentamicin and streptomycin [114].

Beyond matrix components, biofilm development and maturation rely on specific surface-associated proteins. Among these, OmpA appears as a major determinant of biofilm formation, mediating bacterial adhesion to host epithelial cells and abiotic surfaces and contributing to surface motility and immunomodulation [83].

Additionally, BAP, the chaperone-usher pilus system, and Cold-Shock Protein C contribute significantly to the formation and stabilization of these microbial communities [115,116,117,118]. Recently, a polyamine acetyltransferase has also been implicated in modulating biofilm formation [119].

Biofilm formation is tightly regulated by complex signaling networks that control the transition from a planktonic to a sessile lifestyle. Notably, the BfmRS two-component system acts as a key regulator of biofilm formation, surface adhesion, pellicle formation, and resistance to serum-mediated killing. Recent reviews also highlight the pivotal role of Quorum Sensing (QS) in coordinating these processes, regulating the expression of virulence factors through density-dependent signal molecules [80].

## 3. Treatments of *A. baumannii* Infections

### 3.1. Diagnostic Methods for Detecting Antibiotic Resistance: From Classical to Emerging Technologies

The alarming rise in multidrug-resistant *A. baumannii* necessitates not only the development of novel therapeutics but also the implementation of rapid and accurate diagnostic tools. As recently emphasized by the WHO, the successful deployment of new antimicrobial agents must be intrinsically coupled with rapid diagnostic testing to guide appropriate therapy, ensure clinical efficacy, and prevent the selection of new resistance mechanisms. In clinical practice, delayed or inappropriate antibiotic therapy is strongly associated with increased morbidity and mortality, particularly in severe infections caused by CRAB [120].

Traditionally, the detection of antimicrobial resistance has relied on conventional phenotypic methods, such as disk diffusion and broth microdilution, to determine the minimum inhibitory concentration (MIC). Although these approaches remain the gold standard for susceptibility testing, they are labor-intensive, require prior bacterial cultivation, and typically take 18 to 36 h to yield results, thereby delaying targeted therapy [121]. To accelerate detection, conventional molecular assays like PCR and nucleic acid amplification tests have been widely implemented. These methods allow for the rapid detection of known resistance genes directly from clinical samples. However, they are inherently limited to predefined targets and cannot reliably detect novel or uncommon resistance mechanisms, nor do they provide quantitative susceptibility data such as MIC values [122]. This can lead to discrepancies between genotypic predictions and phenotypic resistance profiles.

To overcome the limitations of classical methods, several state-of-the-art technologies are currently utilized in modern clinical microbiology laboratories. Matrix-assisted laser desorption/ionization time-of-flight mass spectrometry (MALDI-TOF MS) has become a routine tool, enabling rapid microbial identification and resistance screening in a few hours at relatively low cost [123]. In parallel, next-generation sequencing and whole-genome sequencing have revolutionized the field by providing comprehensive analyses of the bacterial resistome [124,125]. However, their widespread clinical implantation remains constrained by cost, turnaround time, and the need for bioinformatics expertise.

Flow cytometry is currently being employed as a rapid diagnostic tool to assess bacterial viability and morphological changes under antibiotic stress, providing reliable susceptibility profiles in less than 2 h [126]. Looking ahead, several new and emerging non-conventional diagnostic platforms show great potential for point-of-care testing. Additionally, microfluidics and “lab-on-a-chip” technologies are being developed for rapid, single-cell susceptibility testing by integrating nucleic acid detection and phenotypic tracking within miniaturized, automated platforms [127]. Finally, CRISPR-Cas-based diagnostic systems are emerging as highly sensitive and precise biosensors capable of targeting and identifying specific antibiotic resistance genes with unprecedented accuracy [128]. Despite their promise, many of these technologies remain at the developmental or early clinical validation stage.

Beyond general diagnostic platforms, highly specific rapid tests have been exclusively developed to target *A. baumannii* infections in clinical settings. Isothermal DNA amplification techniques, such as loop-mediated isothermal amplification, bypass the need for thermal cycling. Specific assays can now identify *A. baumannii* and its predominant carbapenemases directly from blood cultures in under 30 min [129]. Multiplex immunochromatographic assays, such as the RESIST-Acineto rapid test developed with research networks like the German Center for Infection Research, provide essential point-of-care detection. These lateral flow assays can quickly identify the most common CRAB, specifically OXA-23, OXA-40, OXA-58, and NDM, in under 15 min directly from bacterial cultures, enabling clinicians to make immediate and targeted treatment decisions [130].

Importantly, the integration of these diagnostic tools into antimicrobial stewardship programs is essential to maximize their clinical impact. Rapid and accurate diagnostics not only improve patient outcomes by enabling timely targeted therapy but also reduce unnecessary broad-spectrum antibiotic use, thereby limiting the selective pressure that drives resistance emergence.

### 3.2. The Challenge: Failure of Conventional Therapies

*A. baumannii* has transformed from a once manageable organism into a formidable healthcare threat, primarily due to its remarkable capacity to acquire resistance mechanisms. Although it accounts for approximately 2% of all healthcare-associated infections in the United States and Europe [131], its burden is substantially higher in Asia and the Middle East, where prevalence rates are nearly doubled [132]. More importantly, an estimated 45% of *A. baumannii* isolates are now classified as MDR, a proportion up to four times higher than that reported for other major Gram-negative pathogens, such as *P. aeruginosa* and *K. pneumoniae*, with MDR rates reaching as high as 70% in regions including Latin America and the Middle East. This alarming global resistance profile contrasts sharply with the 1970s, when clinical isolates were largely susceptible to commonly used antibiotics such as ampicillin, gentamicin, and chloramphenicol. Its emergence as a major nosocomial pathogen in the late 1970s has been largely attributed to the strong selective pressure imposed by the widespread use of broad-spectrum antibiotics in hospital settings [133]. Consequently, MDR, extensively drug-resistant, and even pandrug-resistant strains have rapidly emerged [95]. As resistance to most first-line antibiotics has become widespread, clinicians are increasingly reliant on last-resort agents, particularly colistin and tigecycline, to treat MDR *A. baumannii* infections. Alarmingly, resistance to colistin is now being reported worldwide [6,134], further limiting therapeutic options. In response to this escalating threat, the World Health Organization has classified CRAB as a critical-priority pathogen, underscoring the urgent need to develop novel antimicrobial strategies. Consequently, the failure of conventional antibiotics has prompted the scientific community to explore alternative, non-traditional approaches to combat this pathogen.

### 3.3. Alternatives to Antibiotics

#### 3.3.1. Bacteriophage/Phytoextracts and Essential Oils/Probiotics/Immunotherapies/Drug Repurposing

Bacteriophage therapy has shown promising effectiveness against *A. baumannii*, particularly in combating CRAB strains. Compared to traditional antibiotics, bacteriophages provide several unique benefits: (i) they are highly specific to their target strains, which helps preserve the host’s beneficial microbiome, (ii) they self-amplify at the infection site, and (iii) their ability to kill bacteria is completely unaffected by the pathogen’s existing antibiotic resistance mechanisms [135]. Lytic phages begin infection by attaching specific bacterial surface receptors like LPS, OMPs, or capsular polysaccharides. Once they inject their viral genome and commandeer the host’s machinery for replication, they produce holins and endolysins. These enzymes break down the bacterial inner membrane and cell wall from inside, leading to quick cell lysis and the release of new virions [135,136].

Two lytic phages, ISTD and NOVI, isolated from wastewater, have shown strong antibacterial activity against *A. baumannii*. Notably, a detailed evaluation of phage ISTD revealed a highly significant 3.5-log reduction in planktonic viable bacterial counts and a 2-log reduction in biofilm-associated populations [137]. These phages produce depolymerase enzymes that degrade the exopolysaccharide components of the biofilm matrix, thereby enabling deeper phage penetration and enhancing overall antimicrobial effectiveness. This enzymatic degradation gives phages a significant functional advantage over traditional antibiotics, which typically fail to penetrate the dense biofilm matrix [138]. However, the study also observed subsequent bacterial, indicating that monophage therapy may require further optimization, such as the use of phage cocktails, to limit the emergence of resistance. To address this, combinatorial strategies are increasingly applied; a recent review of clinical cases noted that phage therapy is frequently paired with conventional antimicrobials, with about two of the nine analyzed case studies specifically using phage therapy in combination with antibiotic treatment during and after the viral course [139]. Practical and clinical settings have highlighted the efficacy of phage therapy against challenging *A. baumannii* infections. For instance, in a personalized clinical case, a tailored phage cocktail administered alongside antibiotics and surgical debridement successfully cured a severe, trauma-induced *A. baumannii* osteomyelitis that was unresponsive to traditional treatments [140]. Furthermore, on a broader practical scale, a three-year prospective intervention study demonstrated that environmental decontamination of intensive care units using an aerosolized, preoptimized phage cocktail significantly reduced both the nosocomial transmission and the overall infection rates of CRAB strains [141]. A landmark example of successful human application involved a patient with a disseminated, MDR *A. baumannii* infection who recovered after receiving a personalized phage cocktail intravenously in combination with minocycline, demonstrating the clinical viability of this approach [142].

In parallel, various plant-derived compounds and phytoextracts are being investigated for their activity against *A. baumannii*. Natural substances from *Azadirachta indica* (neem), *Psidium guajava* (guava), and *Aloe vera* have demonstrated biofilm inhibition, disruption of bacterial membrane integrity, and synergistic interactions with antibiotics [143,144,145]. These extracts act by interfering with key proteins involved in adhesion and biofilm maturation, specifically by downregulating the expression of the QS regulators and surface adhesins such as OmpA and the Csu pili system. For instance, neem extract has been shown to reduce biofilm mass and alter QS gene expression, while guava and aloe extracts have enhanced the in vitro potency of conventional antibiotics [143,144]. Expanding beyond plant sources, microbial-derived natural products from bacteria and fungi offer a rich reservoir of novel antimicrobial scaffolds and antibiotics adjuvants [146]. Furthermore, modern methods combining genome mining with chemical synthesis have led to the development of optimized synthetic-bioinformatic natural products capable of completely eradicating MDR *A. baumannii* [147].

Essential oils (Eos) and their major active components have demonstrated potent antibacterial and antibiofilm activities against MDR *A. baumannii*. These volatile compounds mainly disrupt the structure of the bacterial cell membrane. Additionally, recent in vitro studies show that the Eos can reduce the expression of biofilm-related genes and inhibit efflux pump activity, thereby restoring the susceptibility of CRAB strains to standard antibiotics when used in synergistic combinations [148].

Similarly, probiotic therapy has become a promising complementary and preventive approach. Probiotic strains, especially *Lactobacillus* species, show antagonistic effects against *A. baumannii* by competitively excluding it from adhesion sites. Additionally, they produce potent antimicrobial substances. These metabolites significantly hinder *A. baumannii* biofilm formation and alter the local microenvironment, providing a non-antibiotic method to prevent colonization [149,150].

Immunotherapeutic approaches, though still in early development, are also being explored for *A. baumannii*. These include vaccine candidates and monoclonal antibody-based therapies, designed to prevent infection or improve bacterial clearance by the host immune system [151,152]. Experimental vaccines targeting OMP and polysaccharide structures specific to *A. baumannii* have shown partial protection in animal models [152]. Nevertheless, challenges such as antigenic diversity among clinical isolates and inconsistent immune responses continue to hinder translation to clinical use.

Finally, due to the slow pipeline for discovering new antibiotics, drug repurposing has become a popular, quick, and cost-effective alternative. This approach involves finding antibacterial effects in existing, FDA-approved drugs that are not originally antibiotics. Several non-antibiotic medications, including antidepressants (e.g., amitriptyline), antihypertensives (e.g., amlodipine), and anti-inflammatory drugs, have shown significant in vitro activity against CRAB strains. These repurposed drugs often work through mechanisms completely different from those of traditional antibiotics. When used together with conventional antibiotics, these agents often produce strong synergistic or additive effects, which can reduce the required antibiotic dose and help fight multidrug-resistant infections [153,154,155].

Together, these alternative strategies represent promising adjuncts or standalone options for managing *A. baumannii* infections, especially when conventional antibiotics fail. However, limitations such as strain-specific effectiveness, lack of robust clinical validation, and the risk of resistance recurrence underscore the need for integrated, multidisciplinary strategies and continued translational research.

#### 3.3.2. Antimicrobial Peptides

Antimicrobial peptides (AMPs) are a promising class of unconventional therapeutic agents. As essential components of the innate immune system in many organisms, their appeal comes from their potent and rapid bacteria-killing ability. Their main mechanism, the direct disruption of bacterial membranes, offers a key advantage: a lower likelihood of developing traditional resistance compared to conventional antibiotics [103]. The typically cationic and amphipathic structure of AMPs enables them to preferentially target and disrupt the negatively charged OM of Gram-negative bacteria, including *A. baumannii*. While polymyxins (like colistin) are already used clinically, the development pipeline includes several new AMPs and AMP-derived molecules targeting *A. baumannii*: Semi-synthetic polymyxin analogues, such as MRX-8 and Upleganan (SPR-206), have been designed to retain potent membrane-disrupting activity against CRAB while minimizing toxicity, especially nephrotoxicity, compared to their parent polymyxin compounds. Both have successfully finished Phase 1 clinical trials. Novel Cyclic Peptides include OMN6, a synthetic cyclic peptide that is progressing to Phase 2 trials. OMN6 kills bacteria by selectively disrupting the membrane integrity of *A. baumannii*, with a unique mechanism that appears to reduce the risk of resistance development in vitro [76]. These AMPs and their analogues are being developed as new treatments for severe infections like hospital-acquired pneumonia and ventilator-associated pneumonia caused by CRAB. Despite their promising potential, the clinical development of AMPs faces significant challenges that limit their systemic use. Key issues include the stability and degradation, as peptides are highly vulnerable to breakdown by host and bacterial proteases, leading to a very short half-life in circulation [156]. Since a lack of perfect selectivity can result in non-specific interactions with mammalian cell membranes, causing side effects such as hemolysis or, as seen with polymyxins, severe kidney damage [157]. High production costs arise because manufacturing and purifying peptides at scale is complex and expensive, which hampers widespread application. These challenges have fueled efforts to develop more durable synthetic mimics. To address these issues, antimicrobial copolymers have been created, inspired by the amphipathic design of AMPs to mimic their activity while providing improved stability and safety.

## 4. Membrane-Targeting Copolymers: A Novel Arsenal Against *A. baumannii*

### 4.1. Definition and Characteristics

**Figure 8:**
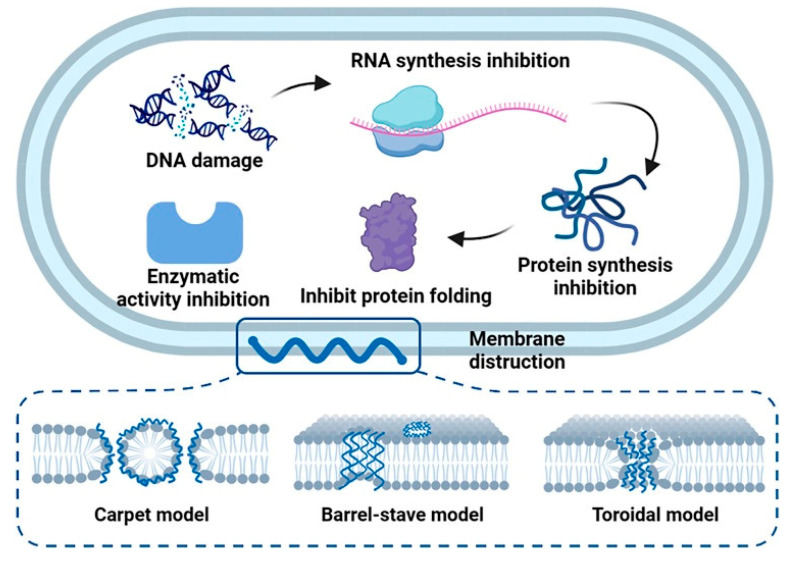
Mechanistic models of membrane disruption were originally developed to describe AMPs, serving as blueprints for synthetic copolymers, figure from [162]. The illustration depicts the classical pathways of membrane permeabilization (e.g., barrel-stave pore, toroidal pore, and carpet mechanisms). Synthetic membrane-active copolymers are engineered to mimic this sequence: (1) initial electrostatic adsorption driven by cationic groups onto the anionic bacterial surface, followed by (2) insertion of hydrophobic segments into the lipid bilayer, leading to (3) structural collapse, loss of transmembrane potential, and cell lysis.

Among the wide variety of polymeric materials, synthetic copolymers have recently gained significant interest because of their potent antibacterial properties. Composed of at least two different monomers, these polymers allow precise tuning of both structural and functional characteristics. In the design of antimicrobial polymers, to mimic AMPs physicochemical properties, the incorporation of cationic and hydrophobic groups is critical [158]. Cationic components, often derived from quaternary ammonium or guanidinium groups, interact electrostatically with the negatively charged bacterial membranes, enhancing selectivity for bacterial cells over mammalian cells due to differences in membrane compositions and charge (i.e., global negative charge of the bacterial membranes and neutral charge of the eucaryotic membranes) [159]. Hydrophobic segments insert into the lipid bilayer, leading to severe membrane disruption. Figure 8 illustrates these well-established mechanisms of action—such as the carpet model or pore formation—originally described for natural AMPs, which synthetic copolymers are specifically designed to replicate. Ultimately, this mechanism bypasses conventional resistance mechanisms, leading to rapid cytoplasmic leakage and bacterial cell death.

Various architectures, including homopolymers, block and random copolymers, have been developed to achieve the optimal balance between hydrophobicity and cationic charge. Amphiphilic block copolymers show excellent ability to target membranes and can self-assemble into micelles, enhancing their effectiveness in aqueous environments [160]. These formations can penetrate biofilms, disrupt bacterial colonies, and deliver antibacterial agents more efficiently. Regarding their antibiofilm activity, they prevented initial surface adhesion while enhancing bacterial surface motility; this dual action effectively prevented biofilm formation in both Gram-positive and Gram-negative pathogens. Crucially, these cationic polymers can exhibit rapid bactericidal kinetics, often eliminating bacteria within minutes through membrane destabilization, a property modulated by their hydrophobic balance. For example, cationic block copolymers made from methacrylate monomers have demonstrated strong bactericidal effects against a broad spectrum of Gram-negative strains, including *A. baumannii*, with MIC values ranging between 7.8–15 µg/mL, and limited to no hemolytic effect (HC_50_ superior to 2000 µg/mL for most of the polymers) giving excellent selectivity indexes (HC_50_/MIC > to 266) [161].

Synthetic copolymers have a key benefit over traditional antimicrobial peptides: enhanced stability. While natural peptides are susceptible to enzymatic degradation and often have short in vivo half-lives, synthetic versions are intrinsically resistant to such protease-mediated breakdowns. Their adaptable backbone allows for modifications in molecular weight, chain rigidity, and segment polarity, which all affect their antimicrobial effectiveness and compatibility with cells. Furthermore, recent developments in controlled polymerization techniques, such as RAFT (Reversible Addition-Fragmentation chain Transfer) and ATRP (Atom Transfer Radical Polymerization), enable the production of highly uniform and reproducible antimicrobial copolymers [163,164].

**Figure 9:**
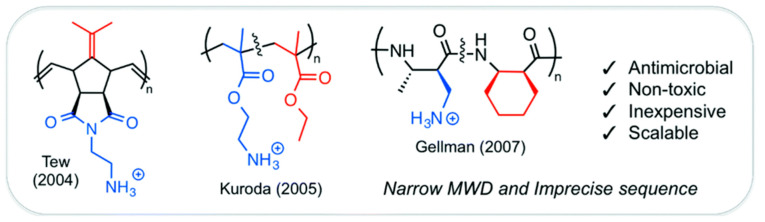
Foundational architectures of non-degradable synthetic antimicrobial copolymers, figure from [158]. The three foundational families of peptidomimetics that proved the concept of synthetic antimicrobial activity are: facially amphiphilic polynorbornenes (Tew, 2004) [167], random polymethacrylates (Kuroda, 2005) [166], and helical polyamides/beta-peptides (Gellman, 2007) [168]. The annotation ‘Narrow Molecular Weight Distribution (MWD) and imprecise sequence’ indicates that these agents combine a controlled chain length (low dispersity) with a random monomer distribution, showing that, unlike natural peptides, a specific amino acid order is not required for activity. These structures show that a specific amino acid sequence is not necessary to develop selective, effective, and scalable antimicrobial agents.

Capitalizing on these synthetic advances, researchers have investigated various backbones to optimize amphiphilicity and activity. To translate these physicochemical principles into functional materials, three main non-degradable architectures were developed: polynorbornenes, polymethacrylates, and polyamides (Figure 9). Tew’s research introduces a polynorbornene created through Ring-Opening Metathesis Polymerization (ROMP), offering a rigid bicyclic framework for precise amphiphilic modifications [165]. Additionally, polymethacrylates provide a scalable and adaptable vinyl-based alternative that can be produced via radical polymerization [166]. Amphiphilic polymethacrylate derivatives are being explored as antimicrobial agents, with MICs ranging from 16 to 60 μg/mL against *E. coli* but with hemolytic concentrations ranging from <1 to 180 μg/mL depending of the percentage of butyl group present in the polymers. Polymers with the highest percentage of butyl group showed the lowest HC_50_ (<1 μg/mL) whereas polymers with 10 to 30% of butyl group were found less hemotoxic with a maximum selectivity (HC_50_/MIC) of 3 at 17% of butyl group.

### 4.2. Non-Degradable Copolymers

#### 4.2.1. Vinyl-Based Copolymers (Poly(meth)acrylates)

The development of synthetic antibacterial poly(meth)acrylates was essential to address the high costs and instability associated with natural AMPs. Unlike peptides, which depend on specific secondary structures such as α-helices for their activity, synthetic copolymers function through their overall amphiphilicity. However, structure–activity relationship (SAR) studies comparing block versus random architectures revealed that a rigid sequence is not strictly necessary for antimicrobial activity [169]. Kuroda and DeGrado showed that randomly distributing cationic and hydrophobic components in these molecules can effectively disrupt bacterial membranes [166].

**Figure 10:**
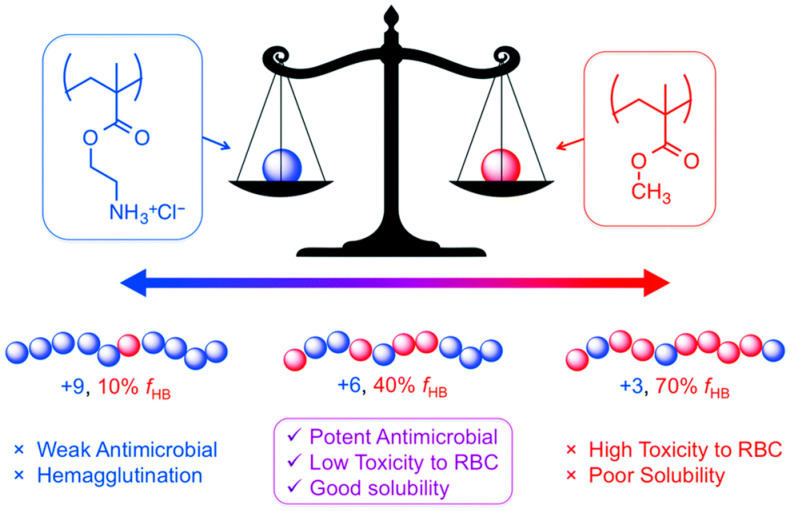
Impact of the amphiphilic balance on copolymer activity and selectivity. Diagram showing the key trade-off between cationic charge (blue) and hydrophobicity (red). (**Left**) Low hydrophobicity (10%) causes weak antimicrobial effects. (**Right**) High hydrophobicity (70%) results in severe hemolytic toxicity to human red blood cells (RBC). (**Center**) An ideal balance (around 40% hydrophobicity) enhances bactericidal activity while maintaining RBC safety, figure from [158].

The effectiveness of these copolymers relies on a precise balance between their hydrophobic and cationic components, referred to as the “amphiphilic balance” (Figure 10). A high density of cationic charges is essential for initial electrostatic binding to the negatively charged bacterial surface. Studies indicate that an optimal composition generally contains approximately 60–70% cationic monomers. However, an excess of cationic groups can reduce antimicrobial activity by hindering the insertion of hydrophobic segments into the lipid bilayer. Hydrophobic groups, such as butyl or phenyl groups, are critical for embedding the bacterial membrane interior. Yet, increasing hydrophobicity beyond an optimal point not only decreases antibacterial effectiveness but also significantly enhances hemolytic activity against human red blood cells, emphasizing the importance of carefully tuning the hydrophobic content for selective antimicrobial action (Figure 10) [170].

Beyond monomer composition, polymer chain length further modulates this selectivity. Molecular weight (Mn), therefore, functions as a selective filter. Kuroda, 2013 [171] showed that copolymers with low molecular weight (<10 kDa) exhibit the most favorable therapeutic window, whereas high-molecular-weight chains (>50 kDa) often lose selectivity and become toxic to mammalian cells due to nonspecific membrane disruption or aggregation [171]. Similarly, the architecture of the polymers, i.e., random or block polymers, affects their toxicity, with block polymers being none hemolytic even at 1000 μg/mL whereas the random polymers show hemotoxicity starting at 1–10 μg/mL.

To improve activity against challenging Gram-negative pathogens such as *A. baumannii*, recent approaches have moved beyond simple ammonium-based antimicrobial polymers.

- Quaternary Ammonium: Common copolymers such as poly(butyl methacrylate)-block-poly(2-(dimethylamino)ethyl methacrylate) (PBMA-b-PDMAEMA), primarily exert their antibacterial activity through membrane disruption and lysis. While these copolymers can be highly effective, their non-specific membrane activity is often associated with significant cytotoxicity, limiting their therapeutic potential [172].
- Guanidinium: In contrast, the incorporation of guanidinium groups markedly improves bacterial selectivity. Unlike ammonium, guanidinium groups can form bidentate hydrogen bonds with phosphate groups present on bacterial membranes. This interaction enhances antibacterial activity with reported minimum inhibitory concentrations (MICs) ranging from 7.8 to 15.6 µg/mL against Gram-negative bacteria, including *A. baumannii* with limited to no hemolytic effect (HC_50_ ≥ 2000 µg/mL) and high selectivity indexes (> to 266) [161].

#### 4.2.2. Current Applications

Vinyl-based antimicrobial copolymers have been predominantly investigated for material applications due to their high chemical stability and robust carbon-carbon backbone, which makes them particularly suitable for permanent antimicrobial coatings on and/or incorporation into biomedical devices. Poly(meth)acrylates are widely used for surface modification of critical medical devices such as catheters, wound dressings, and surgical implants, with the primary goal of preventing biofilm formation, a major cause of hospital-acquired infections. As highlighted by Taresco et al., amphiphilic random copolymers can be designed to inhibit bacterial adhesion and colonization on medical devices, providing broad-spectrum protection against pathogens such as *Staphylococcus* species (MIC = 40 μg/mL) [173]. Through incorporation into materials and surface grafting or coating, these cationic polymers confer so-called “contact-killing” properties, effectively suppressing bacterial attachment and subsequent colonization.

Importantly, specific main-chain cationic polymers developed for surface applications have also demonstrated potent antibacterial activity in solution, exhibiting broad-spectrum efficacy with MICs ranging from 1.5 to 31.25 µg/mL against a panel of MDR ESKAPEE pathogens, including *S. aureus* (MRSA) and *A. baumannii* with no hemotoxicity observed up to 5000 µg/mL giving a high selectivity factor (>160) [174].

### 4.3. The Shift Toward Degradable Copolymers

Despite their potent broad-spectrum antimicrobial activity and low propensity for inducing bacterial resistance, vinyl-based copolymers have seen limited clinical use due to their intrinsic non-degradability of their carbon-carbon backbone. While this structural stability is advantageous for long-term surface coatings and material incorporation, it raises safety concerns for systemic applications: high-molecular-weight polymers that cannot undergo hydrolytic degradation cannot be eliminated by the kidneys and may accumulate in vital organs, risking long-term toxicity. To address these limitations, recent research has shifted toward developing biodegradable polymer structures. The challenge lies in preserving the key “facially amphiphilic” characteristics required for effective bacterial membrane disruption while incorporating cleavable linkages (such as esters, carbonates, or thioesters) within the polymer backbone. These degradable bonds enable controlled breakdown of the polymers into non-toxic, low–molecular–weight fragments that can be efficiently eliminated via renal excretion once their antimicrobial function has been fulfilled.

#### 4.3.1. Biodegradable Micelles

The strategy of self-assembly into cationic micelles has been broadly applied across different polymer chemistries to enhance antimicrobial selectivity; However, when based on non-degradable polymer scaffolds, such systems raise concerns relative to bioaccumulation and long-term toxicity. To mitigate these risks, significant progress has been achieved through the development of biodegradable block copolymers capable of self-assembling into nanostructures such as cationic micelles. These nanostructures concentrate cationic charges at the micellar surface, thereby strengthening electrostatic interactions with negatively charged microbial membranes, while potentially shielding hydrophobic domains until contact with the target cell is made. Nederberg et al. showed that functional cyclic carbonates can be polymerized into amphiphilic block copolymers that self-assemble into cationic micelles in aqueous media, which can selectively disrupt microbial membranes, including those of MRSA, while exhibiting minimal hemolytic activity, highlighting the promise of polycarbonate-based biodegradable micelles [175]. Additionally, Qiao et al. found that antimicrobial effectiveness of these micelles is highly influenced by charge density: an optimal cationic content of approximately 50% was found to maximize activity against Gram-negative bacteria, yielding MIC values of ~4 µg/mL against *E. coli* and ~26 µg/mL against *P. aeruginosa*, whereas a higher charge density (~60%) was more effective against Gram-positive strains [176]. In terms of hemotoxicity, as reported for other types of polymers, polymers with higher hydrophobic content produced more hemolytic activity, but overall, limited hemolysis was observed with this family of polymers, i.e., HC_50_ ranging from 500 to >2000 µg/mL. These findings underscore the critical importance of fine-tuning polymer composition to achieve species-specific selectivity. Unlike traditional antibiotics, they disrupt bacterial membranes without fostering resistance and show strong synergy with imipenem, restoring its effectiveness against resistant strains, indicating a promising direction for combination therapies.

#### 4.3.2. Radical Ring-Opening Polymerization (rROP)

**Figure 11:**
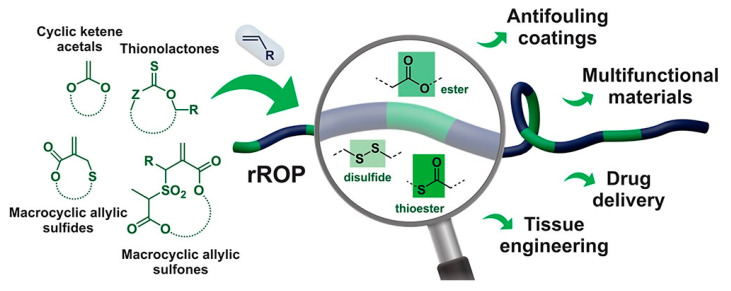
Introducing degradability via rROP from [178] (reproduced with permission). This synthetic approach involves copolymerizing traditional vinyl monomers with cyclic monomers like CKAs or Thionolactones. During radical propagation, the ring-opening mechanism introduces labile bonds such as esters, thioesters, or disulfides into the polymer’s backbone. This makes the polymer biodegradable, suitable for biomedical uses like tissue engineering and drug delivery and helps avoid long-term bioaccumulation.

To overcome the inherent stability of the carbon-carbon backbone in conventional vinyl polymers, rROP has become a key synthetic strategy. This approach combines the robustness and tolerance of radical polymerization with the ability to incorporate heteroatoms, such as oxygen or sulfur, into the polymer backbone—a feature traditionally restricted to ionic or coordination Ring-Opening Polymerization (ROP). This approach enables the integration of degradable ester or thioester bonds into the vinyl polymer backbone (Figure 11), thereby imparting controlled degradability while retaining the advantageous properties of vinyl-derived materials [177,178]. Among the various monomers developed for this purpose, cyclic ketene acetals (CKAs) have proven to be particularly effective. Within this family, 2-methylene-1,3-dioxepane (MDO) remains one of the most widely studied monomers due to its favorable ring-opening behavior.

Unlike conventional vinyl monomers, which polymerize exclusively through carbon-carbon double bond propagation, CKAs undergo a distinct mechanism involving radical addition followed by β-scission of the cyclic acetal ring, resulting in the direct insertion of an ester linkage into the polymer backbone [179]. This mechanism enables the synthesis of aliphatic polyesters, like polycaprolactone, under mild radical polymerization conditions that normally require metal-sensitive catalysts in traditional ROP. The main benefit of rROP is its ability to generate hydrolytically degradable polymers. The ester bonds introduced through monomers such as MDO are susceptible to both chemical and enzymatic hydrolysis, allowing the polymer to degrade into non-toxic, low–molecular–weight metabolites and thereby reducing the risk of long-term bioaccumulation.

Additionally, this method retains the inherent versatility of radical chemistry, enabling the copolymerization of MDO with functional vinyl monomers such as vinyl chloroacetate (VClAc). The inclusion of VClAc is strategic: it provides reactive sites for the introduction of cationic groups. These positively charged moieties are specifically designed to enhance electrostatic interactions with the anionic bacterial membranes of pathogens like *A. baumannii*, creating materials that are both effective and biodegradable [172,177]. A complementary strategy involves creating highly effective, non-degradable vinyl-based copolymers, like polyacrylates, that are suitable for in vivo applications by incorporating sparse “weak links” into their backbone. This concept, known as segmentable copolymers, employs specific cyclic monomers that open their ring structures to form cleavable points. Recent studies have identified thionolactones, in particular dibenzo [c,e]oxepane-5-thione (DOT), as ideal candidates for this approach. Unlike CKAs, which may exhibit unfavorable reactivity ratios with acrylates, DOT copolymerizes efficiently with acrylate and acrylamide monomers [180,181]. Upon ring-opening, DOT inserts a thioester linkage into the polymer backbone, creating predetermined sites that can be selectively cleaved via hydrolysis, aminolysis, or enzymatic degradation [182]. As a result, high-molecular-weight antibacterial copolymers, critical for potent activity against MDR strains, can be subsequently degraded into low-molecular-weight fragments after therapeutic application. Consequently, segmentable vinyl copolymers combine powerful antibacterial properties characteristic of high–molecular–weight vinyl polymers while addressing long-standing concerns regarding toxicity and bioaccumulation [161].

### 4.4. Current Polymers with Efficacy on A. baumannii

An overview of the most effective synthetic copolymers against *A. baumannii*, including their structural properties, minimum inhibitory concentrations (MICs), and in vivo potential, is provided in Table 2 and discussed in detail below. 

A critical analysis of Table 2 reveals that the efficacy of these synthetic polymers against *A. baumannii* is governed by three physicochemical features: cationic charge density (essential for binding to the negatively charged outer membrane), a precisely tuned hydrophilic/hydrophobic balance, and an optimized polymer size to facilitate targeted translocation. Based strictly on the in vitro selectivity index (corresponding to the ratio HC50/MIC), cationic block copolymer methacrylates [161] are highly promising, exhibiting an MIC of 7.8 µg/mL with an HC50 > 8000 µg/mL. However, successful clinical translation against *A. baumannii* requires eradicating established infections in complex environments. Therefore, degradable architectures such as guanidinium-based polycarbonates [183,184] and co-beta-peptides [189], which emerge as the most promising overall candidates. These polymers successfully balance high selectivity with potent anti-biofilm properties and have proven in vivo efficacy in murine wound and lung infection models.

#### 4.4.1. Guanidinium-Functionalized Polycarbonates

A major advancement in antimicrobial polymer design involves incorporating guanidinium groups to mimic arginine residues in host defense peptides. Chin et al. (2018) showed that biodegradable polycarbonates functionalized with guanidinium groups exhibited strong, broad-spectrum activity with MICs of 16 µg/mL against MDR clinical isolates of *A. baumannii*, including strains resistant to carbapenems (imipenem) and last-resort antibiotics like polymyxin B [192].

Unlike quaternary ammonium polymers, which cause membrane lysis, these guanidinium-functionalized polymers operate through a distinct mechanism: they translocate across the membrane and induce precipitation of cytosolic contents. This mode of action is particularly advantageous for systemic infections, as it minimizes the release of endotoxins [183].

Moreover, Ding et al. (2020) [184] demonstrated that these polymers can restore the efficacy of conventional antibiotics while suppressing resistance. When combined with rifampicin, the MIC against MDR *A. baumannii* was reduced by a staggering 2.5 × 10^5^-fold, effectively repurposing the antibiotic. Furthermore, they showed that unlike conventional antibiotics (such as imipenem), which induced a >1000-fold increase in MIC over a 30-day serial passage, the polymer treatment limited this increase to a mere 4–8-fold range [184]. In a complementary approach, Leong et al. (2020) [185] explored polymer-polymer synergy by combining guanidinium and quaternary ammonium oligomers. They demonstrated that this dual-cationic strategy produces a synergistic effect, significantly lowering the MICs of each component required for bactericidal activity [185].

These findings highlight the potential of guanidinium-functionalized biodegradable polymers as a versatile platform for combating MDR pathogens, both as standalone therapeutics and in combination with existing antibiotics.

#### 4.4.2. Self-Assembling Micelles and Nanogels

To overcome the limitations of systemic toxicity and limited bioavailability, self-assembling polymeric systems have been employed to create targeted delivery vehicles. It is important to distinguish between macroscopic hydrogels and nanogels: while both feature crosslinked hydrophilic networks, nanogels are sub-micron-sized particles designed for cellular uptake and systemic circulation. In this context, Zhong et al. (2021) [186] developed cationic micelles (L/D2) capable of encapsulating antibiotics. These nanocarriers exhibited a strong synergistic effect when co-administered with imipenem against MDR *A. baumannii*, reducing the effective antibiotic dose by 4- to 8-fold (FICI < 0.5) and reversing resistance in clinical isolates [186]. For applications requiring localized antibacterial activity, Salek et al. (2021) [193] engineered poly(DMAEMA-co-ethylene dimethacrylate) nanogels via dispersion polymerization. These cationic nanogels interact electrostatically with the negatively charged bacterial membrane, displaying intrinsic bactericidal activity with MICs ranging from 16 to 64 µg/mL against *A. baumannii* strains [193].

#### 4.4.3. Peptidomimetics and Sequence Control

Mimicking natural peptide architecture remains a highly effective strategy for developing potent antimicrobial polymers. Lam et al. (2016) [194] created “star-shaped” polypeptides (SNAPPs) through the random copolymerization of lysine and valine N-carboxyanhydrides. These star polymers exhibited sub-micromolar activity against MDR *A. baumannii* (MICs = 0.6 µM, ≈10 µg/mL), and demonstrated high therapeutic efficacy with negligible toxicity in a murine peritonitis model, outperforming conventional antibiotics [194]. For their part, Luo et al. (2017) developed amino acid block copolymers that combine broad-spectrum antimicrobial activity with specific barrier properties, achieving MICs as low as 32 µg/mL against Gram-negative pathogens [187]. Moreover, Judzewitsch et al. (2018) highlighted the importance of precise sequence control in polymer design, showing that the monomer sequence order in sequence-defined polymers greatly influences antimicrobial efficacy [195].

#### 4.4.4. Advanced Hydrogels and Novel Polymeric Therapeutics

Recent research in antimicrobial materials has moved beyond simple delivery vehicles to explore novel chemical backbones and functionalized topological matrices capable of overcoming the limitations of traditional antibiotics. A notable example is RECCE 327 (R327), a synthetic anti-infective designed to target *A. baumannii* by disrupting cellular energy processes. Unlike classical antibiotics, which act via a “lock-and-key” mechanism on specific proteins, R327 binds to the bacterial OM through hydrophobic interactions. This interaction disrupts the transmembrane potential and depletes cellular ATP, leading to rapid bactericidal effects without inducing resistance. Preclinical studies have demonstrated that R327 is highly effective against MDR strains in wound infection models, offering a promising alternative where conventional therapies fail [50].

In parallel, the development of “smart” materials that respond to the infection microenvironment represents a significant leap forward. Giri et al. synthesized lipoic acid-based, redox-responsive degradable polymers that selectively release antimicrobial agents triggered by elevated concentrations of reducing agents, typical of the intracellular bacterial environment. This targeted release strategy minimizes off-target toxicity while maintaining high local antimicrobial potency [188].

Other strategies incorporate nitric oxide donors into hydrogel networks to trigger biofilm dispersal and sensitize bacteria to treatment. Additionally, Yeo et al. highlighted the potential of contact-killing hydrogels, showing in vivo that physical interactions with bacterial membranes enable rapid biofilm debridement and significantly reduce the burden of MDR *A. baumannii*, confirming their promise for severe topical infections.

For localized treatment, particularly in the context of infected burn wounds, advanced hydrogels have been designed to provide both sustained antimicrobial release and physical bacterial eradication. Atif et al. developed hydrogels functionalized with the synthetic antimicrobial peptide SAAP-148, covalently linking to photocrosslinkable polymers to prevent leaching. These matrices demonstrated broad-spectrum activity and effectively eradicated *A. baumannii* biofilms in skin wound models [190]. Other strategies incorporate nitric oxide donors into hydrogel networks to trigger biofilm dispersal and sensitize bacteria to treatment [196]. Additionally, Yeo et al. highlighted the potential of contact-killing hydrogels, showing in vivo burn-wound models that physical interaction with bacterial membranes enables rapid biofilm debridement and significantly reduces the burden of MDR *A. baumannii*, confirming their promise for severe topical infections [191].

### 4.5. Bypassing A. baumannii Resistance Mechanisms via Engineered Copolymers

The rational design of synthetic antimicrobial polymers is driven by the need to bypass the highly specialized envelope defenses of *A. baumannii* described in Section 2. For example, when *A. baumannii* develops resistance to colistin through the modification or complete loss of LOS, this eliminates the primary binding target of this last resort antibiotic. In contrast, engineered synthetic polymers bypass this mechanism by relying on non-specific electrostatic interactions: their high charge densities and incorporation of highly basic groups, such as a guanidine moiety, enable persistent binding to residual anionic phospholipids independently of LOS presence. Once bound, the finely tuned amphiphilicity architecture of these polymers allows them to intercalate into and physically break apart the lipid bilayer, directly overwhelming the OM barrier typically maintained by the Mla lipid transport system. Additionally, to overcome the protective polyanionic capsule and extracellular polymeric substance in biofilms, which often act as sponges that trap conventional cationic drugs, advanced polymer structures such as PEG micelles are used. These structures partially hide their dense cationic charges during transit, avoiding premature sequestration by capsular polysaccharides, and then undergo local conformational changes to irreversibly disrupt the bacterial membrane.

Collectively, these engineered copolymers bypass multiple resistance mechanisms—target modification, reduced permeability, and extracellular shielding—by exploiting fundamental physicochemical interactions rather than specific molecular targets, thereby reducing the likelihood of classical resistance development.

### 4.6. Limitations of Antimicrobial Copolymers and Emerging Solutions

While antimicrobial copolymers offer significant advantages in fighting resistant pathogens, their clinical and practical use faces multiple challenges. The main issues include direct cytotoxicity to human cells, systemic toxicity affecting organs, particularly nephrotoxicity, limited selectivity, structure–activity trade-offs, and insufficient stability in complex biological environments. Achieving a balance between antimicrobial efficacy and cytotoxicity remains a central issue. Many potent copolymers, especially those with long hydrophobic chains or high cationic charges, can induce hemolysis and other adverse effects on mammalian cells [197,198,199]. Some structural motifs, such as guanidinium-functionalized or multiblock copolymers, show promise for improving selectivity, but their effectiveness is not yet universally [197,200,201]. The hydrophobic/hydrophilic balance is critical: increasing hydrophobicity enhances membrane disruption and antimicrobial activity but also raises cytotoxicity. Similarly, increasing cationic content beyond ≈30% of the molecule generally does not improve antimicrobial effects and may further raise cytotoxicity [199,202]. Another challenge is environmental stability; certain copolymers lose activity in the presence of proteins or under physiological conditions, limiting their in vivo effectiveness [199]. For surface coatings, maintaining long-term antimicrobial activity without releasing toxic fragments remains both an engineering and safety challenge [203]. To overcome these issues, emerging solutions focus on precise molecular design, functional responsiveness, and the incorporation of bio-based components. For example:

- Predictive models based on hydrophobicity indices can be used to optimize the hydrophobic/cationic balance, which can enhance antimicrobial effectiveness while minimizing hemolytic risk [204,205].
- Sequence-controlled and architecture-specific designs, such as co-β-peptides or tapered block copolymers, are being developed to improve bacterial selectivity and reduce mammalian cytotoxicity [189,205].
- Natural or bio-derived monomers, like tropolone-based hydrophobic monomers, offer another approach to enhance biocompatibility without compromising efficacy [204].
- Multifunctional surface coatings combining antimicrobial and antifouling properties, e.g., PEG for antifouling and guanidine for antibacterial action, are under development [173,206,207]. These dual-function coatings help prevent biofilm formation on medical devices and improve their durability.
- For environmental responsiveness, new copolymers are engineered to react to pH, enzymatic activity, or humidity, enabling targeted drug release or activation at infection sites [208,209].
- Cross-linked networks and bottlebrush architectures help sustain antimicrobial activity under physiological conditions and extend coating lifespan [206,209]. Some copolymers, especially those that disrupt membranes non-specifically, show a low likelihood of resistance development, even after repeated exposure [210], making them promising candidates when conventional antibiotics fail.
- Based on the demonstrated synergistic effect of polymers and antibiotics, it will be interesting to design and synthetize chimeric hybrid molecules containing antibiotics covalently linked to the antimicrobial polymers
- Importantly, although in vivo studies have proved that polymers are efficient against bacterial infection, including by *A. baumannii*, pharmacokinetic (PK/PD) studies and ultimately clinical trials will be crucial before being able to use these molecules in veterinary or human medicine.

Overall, although challenges related to toxicity, selectivity, and in vivo stability remain, ongoing research in rational molecular design, bio-responsiveness, and multifunctional systems is paving the way toward safer, more effective, and clinically viable antimicrobial materials.

## 5. Conclusions

The rise in AMR represents one of the most critical public health threats of the 21st century. *A. baumannii* exemplifies this crisis: by leveraging its impermeable OM and ability to form recalcitrant biofilms, it has rendered most conventional antibiotics ineffective. In this context, this review highlights that membrane-active synthetic copolymers have emerged not merely as alternatives but as a breakthrough therapeutic modality.

Unlike traditional antibiotics that are limited to specific “lock-and-key” targets, these copolymers use a nonspecific mechanism of membrane disruption that largely bypasses existing resistance pathways. While early generations of vinyl-based polymers faced challenges regarding bioaccumulation and toxicity, the field has undergone a pivotal shift toward biocompatibility and precision. The development of degradable backbones, via strategies such as rROP and polycarbonate synthesis, now allows for the creation of potent antimicrobial agents that break down into non-toxic metabolites after fulfilling their function.

Translating these innovations into clinical practice faces key challenges. A primary issue is the high potency of these macromolecules, as many current copolymers require MICs that are much higher than those of traditional small-molecule antibiotics. Future studies should aim to optimize the structure–activity relationship, especially by balancing cationic and hydrophobic components, to enable bactericidal effects at lower doses. This adjustment can expand the therapeutic window and reduce systemic toxicity.

The clinical promise of this polymer-based approach is further reinforced by the recent advancement of synthetic anti-infectives into the pharmaceutical pipeline. As demonstrated by candidates like RECCE 327 (R327), which are progressing into clinical stages, this approach validates non-specific membrane disruption via synthetic hydrophobic backbones as a viable therapeutic modality. This breakthrough offers a powerful, translatable solution to combat WHO-priority pathogens that are resistant to traditional antibiotics.

Ultimately, combating *A. baumannii* will require a combination of precise diagnostics, synergistic therapies, and active agents like copolymers. Collaboration among materials science, microbiology, and clinical pharmacology is essential. Increased investment in early-stage research will make interdisciplinary innovation key to improving infection management and restoring our ability to treat bacterial infections effectively.

## Figures and Tables

**Table 1:** List of antibacterial agents being developed against WHO priority pathogens.

Product Name (Code)	Chemical Class	Mode of Action (MoA)	Innovation Status (WHO Criteria)	Phase	Ref.
Apramycin (EBL-1003)	Aminoglycoside	Inhibition of protein synthesis (binds to 30S ribosomal subunit).	Not innovative (known classes/targets).	Phase I	[12,13,14,15,16,17,18,19,20,21,22]
BWC0977	Bacterial topoisomerase inhibitor (NBTI)	DNA Gyrase GyrA and Topoisomerase IV inhibition (DNA replication/synthesis disruption).	Innovative (new chemical class with lack of known cross-resistance).	Phase I	[23]
Cefepime + Zidebactam WCK 5222	DBO-BLI/PBP2 binder + cephalosporin	Cefepime: Cell wall synthesis inhibition (PBP3). Zidebactam: β-lactam enhancer (binds PBP2) and broad-spectrum β-lactamase inhibition.	Not innovative (combination of known classes, though Zidebactam has a novel “enhancer” mechanism).	Phase III	[24,25,26,27,28,29,30,31,32,33,34]
“Funobactam +imipenem + cilastin” XNW4107	“BLI + carbapenem + degradation inhibitor”	Imipenem: Cell wall synthesis inhibition. Funobactam: Broad-spectrum diazabicyclooctane (DBO) β-lactamase inhibitor (Classes A, C, D).	Not innovative (combination of known classes).	Phase III	[35,36]
KSP-1007 + Meropenem	Boronate BLI + β-lactam (carbapenem)	Meropenem: Cell wall synthesis inhibition. KSP-1007: Broad-spectrum inhibition of Serine and Metallo-β-lactamases (MBLs).	Innovative (new chemical class of BLI targeting MBLs).	Phase I	[37]
Meropenem + ANT3310	DBO-BLI/PBP2 binder + β-lactam (carbapenem)	Meropenem: Cell wall synthesis inhibition. ANT3310: DBO-BLI (restoring carbapenem activity against OXA-CRAB and SBLs).	Not innovative (known classes/targets).	Phase I	[38,39]
MRX-8	Polymyxin	Direct membrane effect (disrupts bacterial membranes).	Not innovative (analogue of existing class).	Phase I	[40,41,42,43,44]
OMN6	Insect host defense peptide	Direct membrane effect (selective disruption of bacterial membrane integrity).	Innovative (new chemical class, biological agent).	Phase II	[45,46]
QPX9003	Polymyxin	Direct membrane effect (synthetic polymyxin derivative disrupting the OM).	Inconclusive (or not innovative as it is a derivative of a known class).	Phase I	[47,48,49]
Recce-327 R327	Synthetic (acrolein)polymer	Binds to the OM and disrupts bacterial energy production (ATP), cell growth, and division.	Innovative (new chemical class and MoA).	Phase II	[50,51]
Upleganan (SPR-206)	Polymyxin	Direct membrane effect (disrupts bacterial membranes).	Not innovative (analogue of existing class).	Phase I	[52,53,54,55,56]
Xeruborbactam + beta-lactam (S-649228)	Boronate-BLI + undisclosed IV β-lactam	Broad-spectrum β-lactamase inhibition (serine and MBLs, including KPC, NDM, VIM, OXA-23/48).	Innovative (new chemical class of BLI).	Phase I	[57,58,59,60,61,62,63,64,65]
Zifanocycline (KBP7072)	Tetracycline (aminomethylcycline)	Protein synthesis inhibition.	Not innovative (known class/target; minimal impact of acquired tetracycline resistance).	Phase I	[66,67,68,69,70,71]
Zosurabalpin (RG6006)	Macrocyclic peptide	Inhibits lipopolysaccharide (LPS) transport (disrupts Gram-negative cell membranes).	Innovative (new target/mechanism of action).	Phase I	[72,73,74]

**Table 2:** Summary of the main synthetic copolymers demonstrating efficacy against *A. baumannii.*

Polymer Architecture/Chemistry	Degradability	Antimicrobial Activity on *A. baumannii*	Antimicrobial Activity Other Bacteria (MIC in μg/mL)	Hemolytic Activity HC_50_ (µg/mL)	Antibiofilm Activity on *A. baumannii*	In Vivo Efficacy	Ref.
Cationic block copolymers (Methacrylates)	No	MIC 7.8 µg/mL	*E. coli* (7.8 to 15.6) *P. aeruginosa* (15.6) *S. aureus* (7.8 to 15.6)	170 to >8000	No	No	[161]
Guanidinium-based polycarbonate	Yes	MIC 16 µg/mL	*E. coli* (3.9 to 62.5) *P. aeruginosa* (15.6 to 500) *S. aureus* (7.8 to 1000) MRSA (8 to 16)	62.5 to >8000	No	Yes	[183,184]
QAC & Guanidinium homo/copolymers	No	MIC 7.8 to 15.6 µg/mL	*E. coli* (7.8 to 31.3) *K. pneumoniae* (7.8 to 125) *S. aureus* (3.9 to 7.8)	ND	No	No	[185]
PEG-PGC20-PLLA20/PDLA2 micelles	Yes	MIC 16 to 256 µg/mL	ND	ND	Yes Disruptive at MIC	Yes	[186]
K100L40 block copolymers	Yes	MIC 100 µg/mL	*S. aureus* (100) MRSA (100) *E. coli* (100) *P. aeruginosa* (100) *K. pneumoniae* (100)	ND	No	No	[187]
Disulfide/benzyl lipoate	Yes	MIC 16 to 32 µg/mL	*E. coli* (32 to >256) *P. aeruginosa* (32 to >256)	<125 to >2000	ND	No	[188]
Co-beta-peptides	Yes	Inhibition zone	*E. coli* (16 to 32) *P. aeruginosa* (16 to 32) *S. aureus* (16 to 128) MRSA (16 to 128)	156 to 5000	Yes Disruptive at MIC	Yes	[189]
Poly(HEAAm-co-SAAP-148)	Yes	Contact-Kill	Inhibition zone: *E. coli* *P. aeruginosa* *S. aureus*	ND	ND	No	[190]
Hydrogel	ND	Contact-Kill	Contact-Kill: *E. coli* *K. pneumoniae* *P. aeruginosa* *S. aureus* MRSA	ND	Yes	ND	[191]

ND, not determined.

## Data Availability

No new data were created or analyzed in this study. Data sharing is not applicable to this article.

## Abbreviations

AMR	Antimicrobial resistance
ATRP	Atom Transfer Radical Polymerization
CarO	Carbapenem-associated outer membrane protein
CKAs	Cyclic ketene acetals
CRAB	Carbapenem-resistant *A. baumannii*
DOT	Dibenzo [c,e]oxepane-5-thione
eDNA	extracellular DNA
Eos	Essential oils
LOS	Lipooligosaccharides
LPS	Lipopolysaccharide
MALDI-TOF MS	Matrix-assisted laser desorption/ionization time-of-flight mass spectrometry
MDO	2-methylene-1,3-dioxepane
MDR	Multidrug-resistant
MIC	Minimum inhibitory concentrations
MRSA	Methicillin-resistant *S. aureus*
MWD	Molecular Weight Distribution
OM	Outer membrane
OMPs	Outer membrane protein
OmpA	Outer membrane protein A
PNAG	Poly-β-(1,6)-N-acetylglucosamine
RAFT	Reversible Addition-Fragmentation chain Transfer
rROP	Radical ring-opening polymerization
VClAc	Vinyl chloroacetate
